# Death from the Use of Atropine Drops

**Published:** 1902-06-28

**Authors:** 


### DEATH FROM THE USE OF ATROPINE DROPS.

A case reported to the last meeting of the Ophthalmological Society should inculcate caution in the use of atropine drops in the case of very young children. The patient in this case was a very delicate-looking child, aged 10 months, who had suffered from convulsions most of its life. Three drops of a 1 per cent. solution of atropine were put into the eyes, but they did not dilate the pupils fully. More atropine was then applied, but during the 30 hours which elapsed after the first application of the atropine, only seven drops were used. The temperature, however, rose to 105·6, the pulse became uncountable, and the respirations ran up to 72 per minute. The skin was red, and the lips had a quantity of mucus about them, and in this state the child died. The death was supposed to be due to the atropine.

